# Supplementary material for: Gene Content and Coding Diversity of the Growth Hormone Loci of Apes
Source: Genes (Basel). 2023 Jan 17;14(2):241. doi: 10.3390/genes14020241 (PMC9956162; doi:10.3390/genes14020241)
Supplement: Supplementary file 1 [file genes-14-00241-s001.zip › genes-2027451-supplementary/genes-2027451-SM/Supp Figure 1.pdf]

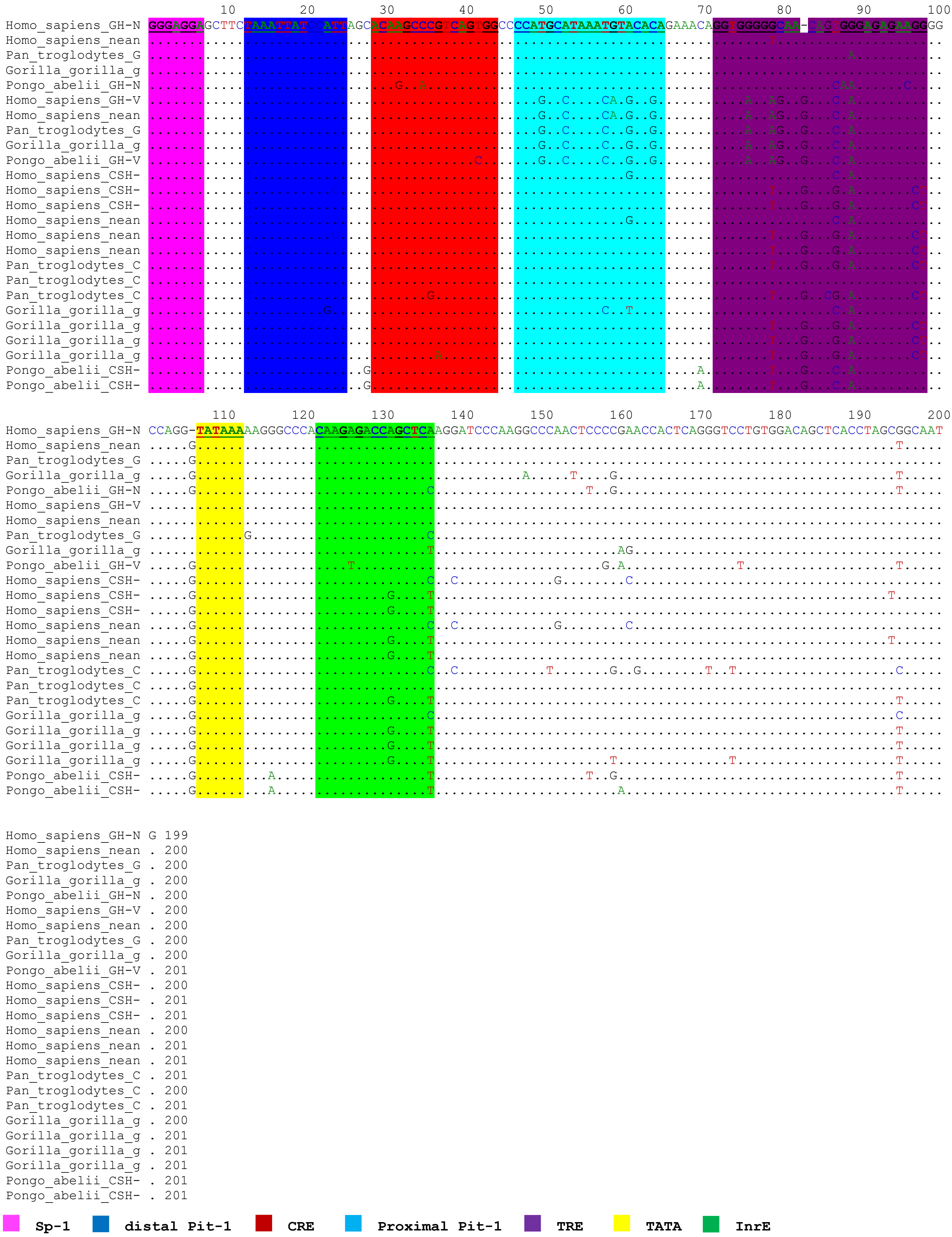

Figure S1 alignments of promoters' nucleotide sequences.
