# Supplementary figures and images for: Gene Content and Coding Diversity of the Growth Hormone Loci of Apes
Source: Genes (Basel). 2023 Jan 17;14(2):241. doi: 10.3390/genes14020241 (PMC9956162; doi:10.3390/genes14020241)

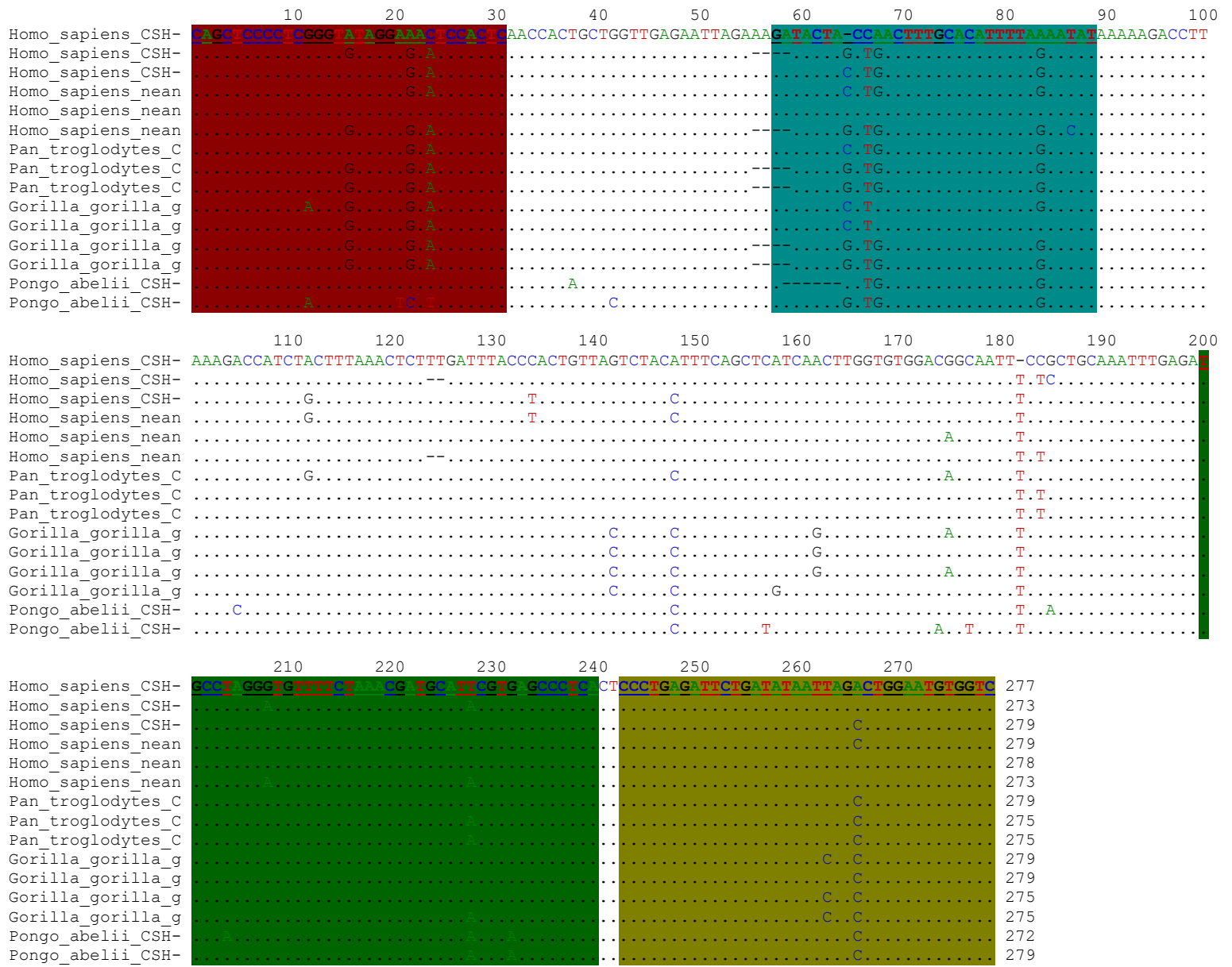

Figure S2 alignments of enhancers nucleotide sequences

Supplement: Supplementary file 1 [file genes-14-00241-s001.zip › genes-2027451-supplementary/genes-2027451-SM/Supp Figure 2.pdf]
